# Improving Decision making On Location of Care with the frail Elderly and their caregivers (the DOLCE study): study protocol for a cluster randomized controlled trial

**DOI:** 10.1186/s13063-015-0567-7

**Published:** 2015-02-12

**Authors:** France Légaré, Nathalie Brière, Dawn Stacey, Henriette Bourassa, Sophie Desroches, Serge Dumont, Kimberly Fraser, Adriana Freitas, Louis‐Paul Rivest, Lise Roy

**Affiliations:** Research Centre of the CHU de Québec, St-François D’Assise Hospital, 10, rue de l’Espinay D6-735, Quebec City, G1L 3 L5 Canada; Department of Family Medicine and Emergency Medicine, Faculty of Medicine, Université Laval, 1050, avenue de la Médecine, Quebec City, G1V 0A6 Canada; Centre de santé et de services sociaux (CSSS) de la Vieille-Capitale, 880, rue Père-Marquette, Quebec City, G1M 2R9 Canada; Ottawa Hospital Research Institute, 501 Smyth Road, Ottawa, K1H 8 L6 Canada; School of Nursing, University of Ottawa, 451 Smyth Road, Ottawa, K1H 8 M5 Canada; Caregivers’ representative, Research Centre of the CHU de Québec, St-François D’Assise Hospital, 10, rue de l’Espinay, Quebec City, G1L 3 L5 Canada; Department of Food Science and Nutrition, Université Laval, 2425 rue de l’agriculture, Quebec City, G1V 0A6 Canada; School of Social Work, Université Laval, 1030, av. des Sciences-Humaines, Quebec City, G1V 0A6 Canada; Faculty of Nursing, University of Alberta, 11405 87 Avenue, Edmonton, T6G 1C9 Canada; Faculty of Sciences and Engineering, Department of Mathematics and Statistics, Université Laval, 1045 rue de la médecine, Quebec City, G1V 0A6 Canada

**Keywords:** Elderly, Home care, Shared decision making, Interprofessional, Implementation, Knowledge translation, Training, Ottawa decision support framework, Decisional conflict scale, Cluster randomized trial

## Abstract

**Background:**

One of the toughest decisions faced by elderly people is whether to stay at home or move to a care facility. This study seeks to evaluate the impact of training interprofessional home-care teams in shared decision making combined with a decision aid on the proportion of elderly people who report being active in the decision-making process regarding whether to stay at home or move to a care facility.

**Methods/Design:**

We propose a multicenter cluster randomized trial conducted with home-care interprofessional teams in the Province of Quebec with 2 data collection phases: before and after the intervention. Units of randomization will be centers for primary healthcare and social services. We will enroll 16 of these and ask each to provide one home-care interprofessional team involved in decisions and care planning with eligible clients. Clients will be included if they i) are aged ≥65; ii) are receiving care from the participating home-care interprofessional team; iii) have faced the decision about staying at home or moving to a care facility in the past 3 to 6 months; iv) are able to read, understand and write French or English; and v) are able to give informed consent. If clients are unable to provide informed consent, their primary caregiver who was involved in the decision-making process will be eligible to participate. The intervention arm will receive training in shared decision making and use of a decision aid. The control arm will receive ‘usual care’. The primary outcome of interest is the assumed role in the decision-making process as assessed in clients or caregivers with a modified version of the Control Preferences Scale. Multilevel modeling will be used to take the hierarchical structure of the data into account. The study has obtained full ethical approval. The trial will comply with CONSORT guidelines adapted for cluster randomized trials.

**Discussion:**

Home care is a rapidly growing sector and this study will lay the foundations of a national strategy to ensure that IP home-care teams provide the highest quality of care for seriously ill elderly people and support for their families.

**Trial registration:**

ClinicalTrials.gov NCT02244359 (registered 18 September 2014).

**Electronic supplementary material:**

The online version of this article (doi:10.1186/s13063-015-0567-7) contains supplementary material, which is available to authorized users.

## Background

Striving to provide the right care in the right place and at the right time for an aging population requiring care is a priority issue for most ministries of health across Canada [1,2]. One of the toughest decisions for elderly people in Canada is whether to remain at home (with or without assistance) or move to a residential care facility [3]. Because this decision is rarely clinically cut-and-dry and is highly preference-sensitive [4,5], it requires shared decision making (SDM) [6]. Shared decision making, a process whereby health professionals and clients work together to make healthcare choices, is fundamental to informed consent and client-centered care [7-10]. Given the emphasis on integrated healthcare services and engagement of clients as partners in their care, finding effective ways to involve clients in sharing decisions with healthcare teams is critical [11-13]. An interprofessional (IP) approach to SDM (IP-SDM approach) is especially relevant to home-care teams working with elderly people. IP-SDM enables IP teams to support them in facing decisions, meet their decisional needs, and reach healthcare choices that are agreed upon by the client, family members/caregivers and the IP team together [14-16]. Since 2007, our team has worked toward expanding SDM beyond the physician-client dyad to IP teams [17] while aiming for rigorous evaluation of interventions for implementing SDM in clinical practice [18]. A previous study found that, overall, home-care providers intend to engage in an IP-SDM approach with elderly people but that various barriers interfere with them doing so [19]. We therefore developed a training program in SDM [20] combined with a decision aid and piloted these with one IP home-care team in Quebec City and one in Edmonton. However, we did not evaluate its impact on clinical practices nor on clients and their caregivers. Therefore, in this study, we seek to evaluate the impact of training IP home-care teams in SDM combined with a decision aid on the proportion of elderly people who report being active in the decision-making process regarding whether to stay at home or move to a care facility.

## Methods/Design

### Trial design and setting

**Figure 1:**
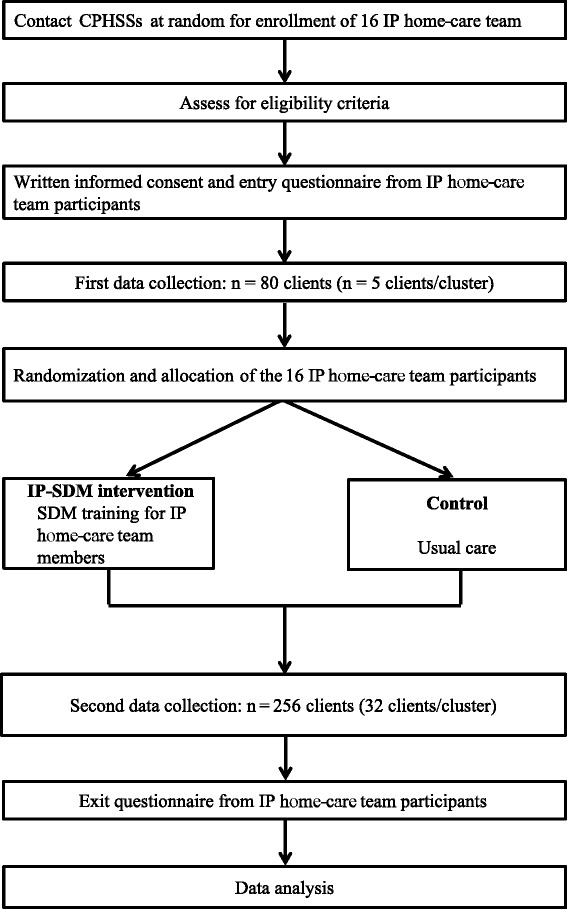
**Study flow diagram.** CPHSS, center for primary healthcare and social services; IP, interprofessional; IP-SDM, interprofessional approach to shared decision making.

We propose a multicenter cluster randomized trial conducted with home-care IP teams in the Province of Quebec and with 2 data collection phases: before (pre) and after (post) the intervention (Figure 1). The unit of randomization will be the center for primary healthcare and social services (CPHSS). CPHSSs (known in Quebec as *Centres de santé et de services sociales*) are the result of mergers between local community service centers, long-term care facilities and, in most cases, a hospital. The CPHSS is accountable for providing the local population with accessibility, continuity and quality of care. Our proposed study design decreases the potential of contamination bias since our proposed intervention targets one home-care IP team in each eligible CPHSS. During 2 data collection phases (pre- and post-intervention), we will assess the outcomes detailed below. Clients and caregivers will not be the same for pre- and post-intervention data collections, but IP teams and providers will be the same. Ethics committee review approval has been obtained from the CHU de Québec multicenter ethics committee (approval number: MP-CHU-QC-14-001). This trial is registered at clinicaltrials.gov (registration number: NCT02244359).

### Engagement with and inclusion of clients, their families and caregivers

The proposed project builds on a long history of successful partnerships with clients’ and caregivers’ representatives. The IP-SDM conceptual model [15,16,21], the decision to be addressed and the training in SDM combined with a decision aid [22] to be evaluated were all identified and developed with stakeholders. Moreover, we have involved 2 caregivers’ representatives (LR, HB), one of whom is a representative of a nonprofit organization who is dedicated to representing caregivers in the writing of this proposal. HB reviewed all questionnaires to ensure their acceptability by clients and caregivers.

This project builds on previous successful client/caregiver partnerships as well as on successful relationships with healthcare organizations such as the CPHSS. An advisory board including representatives of all stakeholders is providing guidance throughout the project.

### Inclusion/exclusion criteria for participants and recruitment

We will target all CPHSSs in the eastern part of the Province of Quebec [23]. We will contact the directors of all potentially eligible CPHSSs in a random manner. We will present the project and seek their interest in participating in the study. One home-care IP team who is involved in decisions and care planning with eligible clients in participating CPHSSs will be eligible to participate in the trial. An IP healthcare team refers to the presence of a minimum of 2 healthcare providers from different professions involved in client care and who collaborate to provide integrated and cohesive client care [24,25]. Clients will be included if they i) are aged ≥65 years; ii) are receiving care from the home-care team; iii) have faced the decision about whether to stay at home or move to a care facility in the previous 3 to 6 months; iv) are able to read, understand and write French or English; and v) are able to give informed consent. For clients who cannot provide informed consent, we will identify the primary caregiver who was involved in the decision of interest. Clients will be recruited by the research team in a consecutive manner. We will keep detailed information on the flow of clients throughout the trial. All participants (providers, clients and caregivers) will sign consent forms approved by the ethics boards of the 16 CPHSSs (see detailed list, Additional file 1).

### Intervention arm

This intervention will include training in SDM and a decision aid. More specifically, the training in SDM will include i) a 1.5-hour online tutorial, based on the Ottawa Decision Support Tutorial [26], and ii) a 3.5-hour skills building workshop, which includes a video that demonstrates SDM in the context of IP home-care teams. Providers will be asked to give clients and their caregivers a copy of the decision aid. The intervention will be staggered over a 6-month period.

### Control/usual care arm

The providers and clients from the control group will not receive any particular intervention apart from the completion of data collection forms. We call the control group ‘usual care’ because clients from the control group will be exposed to the standard procedure for decisions regarding location of care currently in use in the Province of Quebec. This procedure will also be used in the intervention group.

### Allocation of participants to trial groups

Potential confounding variables will be controlled by randomization. The unit of randomization will be the CPHSS with stratification by urban or rural setting [27]. For CPHSS enrollment, 2 random lists will be prepared to establish the order in which CPHSSs will be contacted. We will follow this order systematically until the enrollment of 4 urban and 12 rural CPHSSs is complete.

A second randomization will occur after participants have completed their baseline measures. This randomization will be stratified to minimize the differences within groups due to geographic location. Thus, 2 urban and 6 rural CPHSSs will be allocated to each group. Randomization and allocation will be done using computer software, and data will be entered by an experienced, independent biostatistician.

### Protecting against sources of bias

Given the nature of this trial, the investigators, research assistants (who will enroll participants) and participating CPHSSs and their IP home-care teams will not be blinded to group allocation because they will be aware of which CPHSSs are receiving the intervention. However, sources of bias will be minimized by the following procedures:

1. Strict allocation concealment will be respected by using standardized tools and by instructing research assistants not to tell clients to which group their IP home-care team has been randomized. Moreover, each research assistant will collect data only on the intervention group or only on the control group; they will not change trial arms.
2. The design of data collection forms and packages will be similar in both groups.
3. Double data entry will be centrally performed by 2 independent data clerks who will attribute a secret group code to intervention and control groups and will thus be blinded to group allocation.
4. Data analyses will be performed blindly by our study biostatistician who will not be involved with randomization.
5. Team members will access the group code only after analyses and interpretation of the results are completed. Recruitment of clients will be sequential and will be performed by a research assistant who will be given names of potentially eligible clients directly from a central registry that is in place in all CPHSSs. She/he will then approach all potentially eligible participants consecutively, thus reducing selection bias. We do not expect that providers in the participating CPHSSs will influence one another regarding the intervention, particularly given the significant geographical distance between IP home-care clinical sites. Sociodemographic data in each group will be compared to assess confounding variables.

### Proposed frequency and duration of follow-up

There will be 2 data collection phases: 1) the pre-intervention data collection, lasting 3 months (for a sample of 5 clients/CPHSS), will be used to compare CPHSSs (intervention versus control) on the basis of the CPHSS clientele profile on the proportion of clients reporting an active role in the decision-making process about whether to remain at home or move to a care facility; and 2) postintervention, lasting 10 to 12 months.

### Outcome measures

The primary outcome of interest is the assumed role in the decision making. We will use the modified version of the Control Preferences Scale [28] designed to assess the assumed role in the decision-making process reported by the client or the caregiver [29]. The scale consists of a single question to assess the client’s perception of locus of control over the decision-making process. This is the scale used most in studies assessing the implementation of SDM in clinical practices [18,30]. It is responsive to change and correlates with patients’ as well as with clinicians’ reported levels of patient involvement [31]. Response options (n = 5) are as follows: A) I made the decision; B) I made the decision after seriously considering my providers’ opinions; C) My providers and I shared the responsibility for the decision making; D) My providers made the decision after seriously considering my opinion; E) My providers made the decision. A and B represent a client-controlled decision-making process, C represents a shared decision-making process, and D and E represent a practitioner-controlled decision-making process [29]. We will combine A, B and C to identify the proportion of clients reporting an active role in the decision-making process, and we will combine D and E to identify a passive role. We will perform our analysis by assessing the increase in the percentage of clients reporting an active role.

Measures of secondary outcomes will be collected. More specifically, in clients and caregivers, we will assess their preferred and chosen option (remain at home or move to care facility); decisional conflict using the Decisional Conflict Scale [32,33]; decisional regret using the Decisional Regret Scale [34]; HR-QoL (only in client) using 2 subscales from the Nottingham Health Profile: Social Isolation and Emotional Reactions [35-38]; and burden of care (only in caregivers) using a validated scale [39-42].

In providers, at study entry and exit, we will assess their intention to engage in SDM behaviors in the context of an IP home-care team sharing the decision with elderly people about whether to stay at home or move to another location.

### Statistical consideration

#### Sample size

The primary outcome of interest is the proportion of elderly people who report an active role in the decision-making process regarding whether to stay at home or move to a care facility. Our estimate of the sample size is based on our updated reviews of interventions to improve the uptake of SDM [43] and earlier studies in primary care, in which we observed a 20% absolute difference between groups with an expected baseline value of 50% for the active role [44]. Our biostatisticians provided a number of scenarios with ICCs ranging from 0.02 to 0.05 and the number of clusters ranging from 12 to 16. We used a conservative value of ICC of 0.05 as the upper limit. In order to detect a difference of 20% (50% to 70%) between the intervention group and the control group after the intervention with 80% power, and a 5% significance level, we would require a total of 186 clients for an individually randomized trial. In considering an ICC of 0.05 and 16 clusters (CPHSSs), we would need a sample size of 456 clients in total. Allowing for a 10% loss to follow-up, 501 clients will be recruited (32/CPHSS). We will also recruit 5 clients/CPHSS before the intervention. This is deemed a sufficient number to be able to compare CPHSSs (experimental versus control) at trial entry on the basis of their clientele profiles [45].

#### Analysis plan

We will perform a descriptive statistical analysis of organizational (CPHSS/health professionals) and socio-demographic (clients/health professionals) characteristics in order to assure the comparability of the intervention and control groups. Potentially confounding variables, if any, will be introduced as covariates in mathematical modeling analysis. Multilevel modeling will be used to take the hierarchical structure of the data into account by specifying random effects at each of the 2 levels: 1) CPHSS and 2) client. For each outcome analyzed, according to the type of variable (continuous or categorical), the goodness of fit and the assumptions of each model will be assessed. Statistical analysis will be performed using the SAS statistical package [46]. The primary outcome, i.e. the proportion of clients reporting an active role in the decision-making process, will be analyzed through multilevel logistic regression using the GLIMMIX procedure of the SAS program. The least squared mean proportions will be estimated and compared to assess the effect of the intervention on the primary outcome. Secondary outcomes will be comparisons between the 2 groups (intervention, control) using multilevel modeling. The *P* value will be adjusted for any multiple comparison tests. Adjustment for multiple comparisons will only be made for secondary outcomes, using the Bonferroni method. This will avoid significant results being due to chance alone. Analysis will be performed after the pre-intervention data collection (for providing performance feedback during the workshop) and at the end of the trial. We will explore the impact of differences between male and female clients on the primary outcome.

### Compliance

We will reinforce compliance with the intervention by i) sending reminders before the training in SDM; ii) providing interactive workshops in each of the participating IP home-care clinical sites at a time convenient for providers; iii) offering continuing professional development credits; and iv) monitoring the number of participants accessing and completing knowledge scores in the online tutorial. Compliance with correct completion of questionnaires will be reinforced by detailed instructions provided by the research assistant beforehand and verification of the completed questionnaires by the research assistant afterwards. We will gather detailed information on the flow of participants throughout the trial.

### Losses to follow-up

We expect few losses to follow-up in this trial due to the stability of the population studied, only one data collection point for clients and caregivers, and additional measures to minimize loss. All study participants will be included in the analysis as part of the group to which they were randomized, regardless of whether they completed the study or not (‘Intention-to-treat’ analysis). ‘Worst-case scenario’ sensitivity analysis will be performed for missing data on clients.

### Trial steering committee

The trial steering committee will include all co-principal investigators (FL, DS and NB), the project coordinator (AF), a biostatistician and one caregiver representative. Given the integrated knowledge approach underlying this trial and the nature of the trial, we do not plan to have a data monitoring committee. Research assistants will be instructed to report any adverse events or unintended effects of the trial interventions or trial conduct.

### Dissemination policy

All stakeholders have been directly involved in designing our IP-SDM model and intervention and providing feedback to make them more relevant to the home-care setting. All team members will contribute to the dissemination of study results. We will tailor effective knowledge translation strategies for each targeted users’ group (for example, policy makers, clinicians, healthcare organization managers, seniors’ associations). We will disseminate study results a) at conferences (scientific and professional) whose themes relate to SDM, IP and health policy; b) on the websites of team members; and c) as articles in peer-reviewed journals and professional journals.

## Discussion

We have described the methods that we will use to evaluate the impact of training interprofessional home-care teams in shared decision making combined with a decision aid on the proportion of elderly people who report being active in the decision-making process regarding whether to stay at home or move to a care facility. Results from this trial will lay the groundwork for a national strategy regarding the improvement of the decision-making process for the significant numbers of aging Canadians who are facing a potential transition to care facilities and for their caregivers. Furthermore, skills gained by participants in the intervention arm of this study are likely to be transferable to supporting clients who are making other decisions, such as those relating to mental health. Thus, the results of the proposed trial will concretely address identified challenges and knowledge gaps: 1) the growing number of aging Canadians facing the decision regarding the location of care who need client-centered decision support; 2) the need for improvement of the decision-making process regarding the location of care; 3) the need for a framework to guide IP home-care teams in this process; and 4) the need for training in this process [47]. This project will help IP home-care teams in providing the highest quality of care for seriously ill elderly people and in providing support for their families.

## Trial status

Client recruitment started on 14 October 2014, and we anticipate recruitment will be completed in March 2016.

## Additional file

Additional file 1:
**List of the ethics boards for the 16 centers for primary healthcare and social services (CPHSS).**

## Abbreviations

CPHSS: center for primary healthcare and social services
CSSS: Centre de santé et de services sociaux
IP: interprofessional
IP-SDM approach: interprofessional (IP) approach to SDM
SDM: shared decision making
