# Supplementary material for: Improving Decision making On Location of Care with the frail Elderly and their caregivers (the DOLCE study): study protocol for a cluster randomized controlled trial
Source: Trials. 2015 Feb 12;16:50. doi: 10.1186/s13063-015-0567-7 (PMC4337186; doi:10.1186/s13063-015-0567-7)
Supplement: Additional file 1: — List of the ethics boards for the 16 centers for primary healthcare and social services (CPHSS). [file 13063_2015_567_MOESM1_ESM.doc]

List of the ethics boards (CÉR, or Comité d'éthique de la recherché) of the 16 CPHSSs. Please note that some ethics boards are not mutually exclusive because they cover more than 1 CPHSS.

CÉR du CSSS de la Vieille-Capitale for:

- CSSS de la Vieille-Capitale
- CSSS de Québec-Nord
- CSSS de Portneuf

CÉR du CSSS de Rimouski-Neigette

CÉR du CSSS de Chicoutimi for:

- CSSS de Chicoutimi
- CSSS de Jonquière
- CSSS du Lac-Saint-Jean-Est

CÉR du CSSS de Beauce

CÉR du CSSS de Rocher-Percé

CÉR du CSSS de Trois-Rivières

CÉR du CSSS Alphonse-Desjardins for:

- CSSS Alphonse-Desjardins
- CSSS de Montmagny-L’Islet

CÉR du CHU de Québec (MP-CHU-QC-14-001) for:

- CSSS de La Matapédia
- CSSS de Kamouraska
- CSSS de Maskinongé
- CSSS de Charlevoix
- CSSS Haute-Côte-Nord-Manicouagan
